# Exploring emphysematous osteomyelitis as a potential cause of severe unexplained back pain in advanced metastatic cancer patients

**DOI:** 10.1016/j.radcr.2024.09.028

**Published:** 2024-09-26

**Authors:** Ahmad Alelaumi, Ismail Althunibat, Almu'atasim Khamees, Mohammad Alfawareh, Osama Khalil

**Affiliations:** a Department of Orthopedics and Spine Surgery, King Hussein Cancer Center, Amman, Jordan; b Department of Surgery, King Hussein Cancer Center, Amman, Jordan; c Princess Basma Teaching Hospital, Ministry of Health, Irbid, Jordan

**Keywords:** Emphysematous osteomyelitis, Gas-forming bacteria, Radiology, Spine, Back pain

## Abstract

Emphysematous osteomyelitis (EO) a rare and potentially life-threatening condition caused by gas-forming bacteria, can occasionally affect the spine. Timely diagnosis and appropriate intervention are crucial in preventing rapid deterioration. This article aims to provide insights into the clinical presentation and radiographic findings of emphysematous osteomyelitis of the spine in advanced metastatic cancer patients who may present with severe, unexplained back pain. We present 2 cases of emphysematous osteomyelitis in patients with advanced metastatic cancer. The first case was for a 37-year-old female and the second case was for a 66-year-old male. In both instances, the diagnosis was established based on distinctive radiographic findings. In both cases, the infection was caused by an unusual organism. Medical treatment resulted in pain management and an improvement in performance status for both patients. In conclusion, Emphysematous osteomyelitis (EO) is a rare condition that necessitates prompt diagnosis. The preferred imaging modalities for diagnosing EO are Computed Tomography (CT) rather than Magnetic Resonance Imaging (MRI). Therefore, a comprehensive radiological assessment is crucial in suspected cases. Early identification and treatment can significantly reduce morbidity in this vulnerable patient population and may improve prognosis.

## Introduction

Emphysematous osteomyelitis (EO) is an infrequent, highly aggressive, and potentially lethal type of infection triggered by bacteria that produce gas. The characteristic feature observed in cases of EO is the detection of gas while ruling out alternate causes such as surgical procedures or penetrating injuries [1,2]. Although the existing literature lacks clarity on whether medical or surgical intervention is more effective, adopting a multidisciplinary approach, particularly in cancer patients, can facilitate early detection and help prevent complications. This article describes a rare occurrence of emphysematous osteomyelitis (EO) in the spine, which was observed in 2 patients with advanced metastatic cancer that had metastasized to the bones. In the first case, EO was caused by Salmonella subspecies, while in the second case, it was caused by Staphylococcus Saprophyticus. The main objective of this report is to provide an overview of the symptoms, associated medical conditions, radiographic findings, treatment approach, and ultimate outcome of this specific type of osteomyelitis.

## Cases presentation

### Case 1

The first case presented is about a 37-year-old female patient who had advanced metastatic breast cancer affecting various sites, including the bones, lymph nodes, and brain. The patient was undergoing weekly chemotherapy and immunotherapy treatments. Upon admission, she complained of mild to moderate upper thoracic back pain. She had experienced chills at home and developed a fever upon arrival at the emergency room. Significantly, the patient had undergone a previous hospitalization 20 days before the current presentation, during which she received a high dosage of steroids.

The patient's condition was concerning, as she exhibited signs of distress with an elevated pulse rate of 167 beats per minute and a blood pressure of 100/66 mmHg. Laboratory results indicated the presence of anemia, with a hemoglobin (Hb) level of 7.3 g/dL (normal range: 12-16 g/dL). The patient's white cell count was measured at 4000 cells/microliter (normal range: 4000-11000 cells/microliter), and the basic metabolic panel (BMP) did not reveal any significant abnormalities. However, the C-reactive protein (CRP) level was significantly elevated, measuring 291 mg/L (normal range: less than 8-10 mg/L). Blood and sputum samples were collected for cultures, and the patient was initiated on a combination of piperacillin/tazobactam and levofloxacin while awaiting the culture results.

The blood culture results indicated the presence of Salmonella enterica subspecies. Accordingly, the patient's antibiotic treatment was adjusted based on the sensitivity results. Five days after admission, the patient was switched to ceftriaxone according to the culture sensitivity findings.

**Fig. 1:**
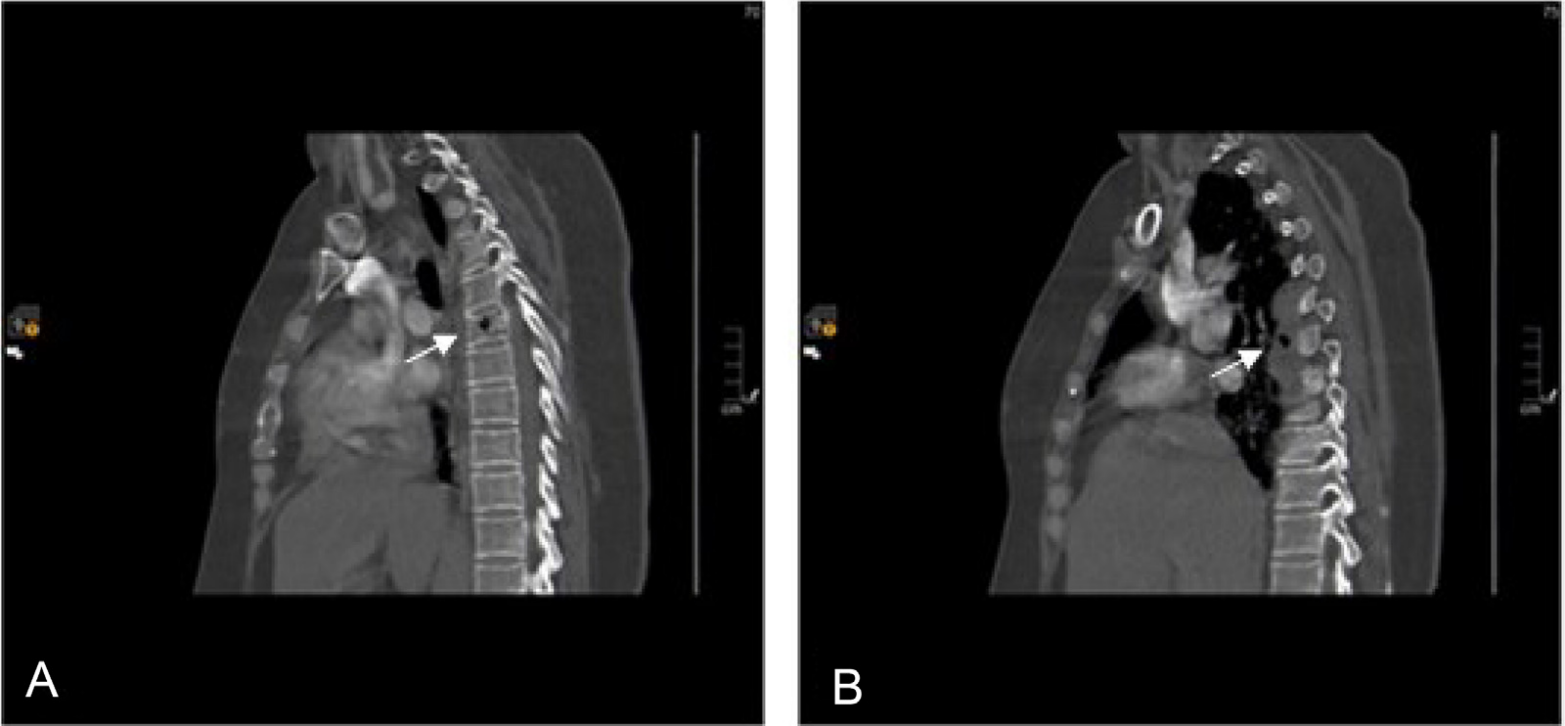
(A) sagittal view of the thoracic spine showing air locules inside the T6 vertebral body (white arrow). (B) sagittal view of the thoracic spine lateral to the previous slide showing a paravertebral collection with air locules extending in the right paravertebral region of T5-T7 levels (white arrow).

**Fig. 2:**
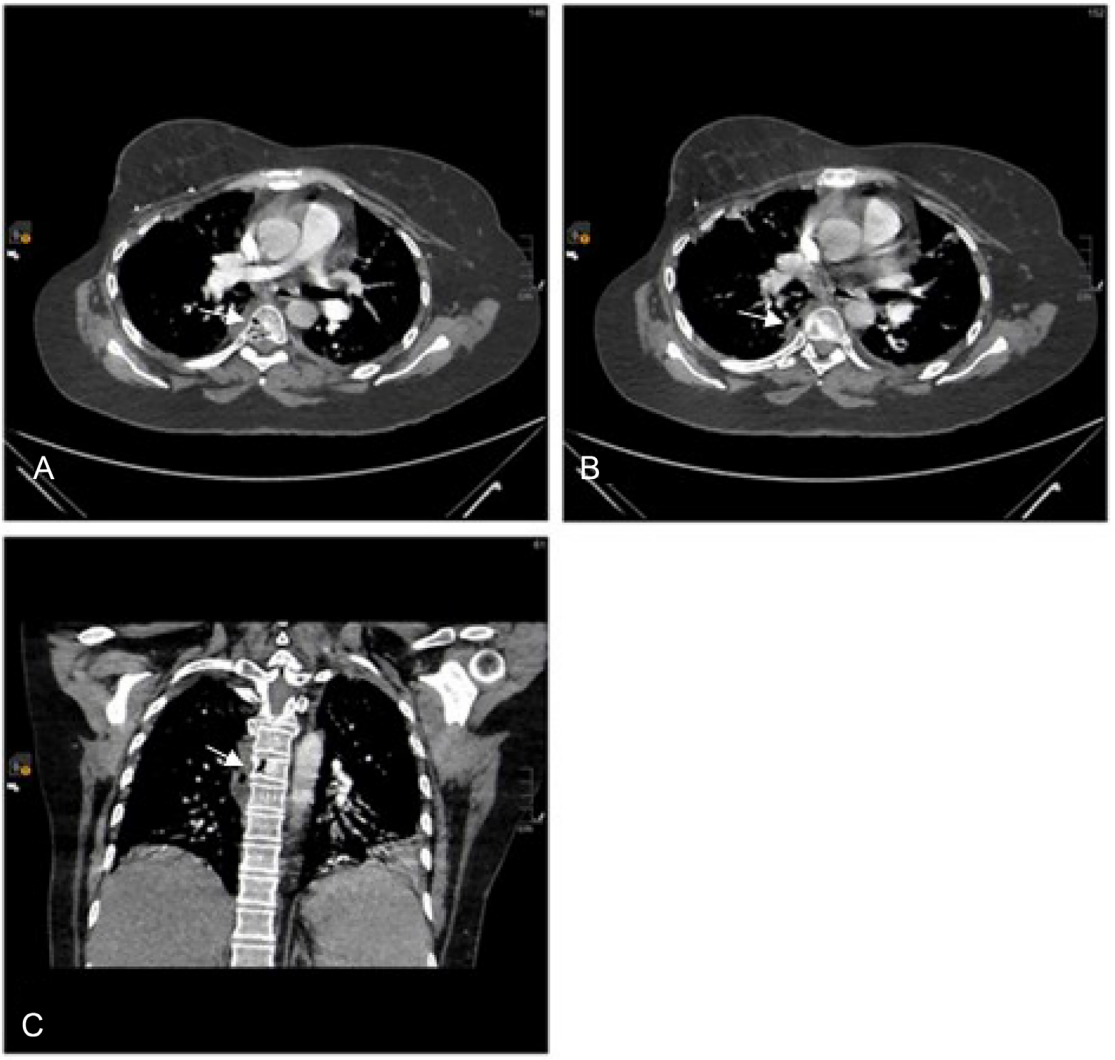
(A) and (B) axial view of the thoracic spine showing intraosseous air locules inside the T6 vertebral body associated with right paravertebral soft tissue fluid collection measuring 3.7×1.5 cm in axial dimensions with extension of the air locules into the right paravertebral region. (C) coronal view showing the same findings (white arrows).

**Fig. 3:**
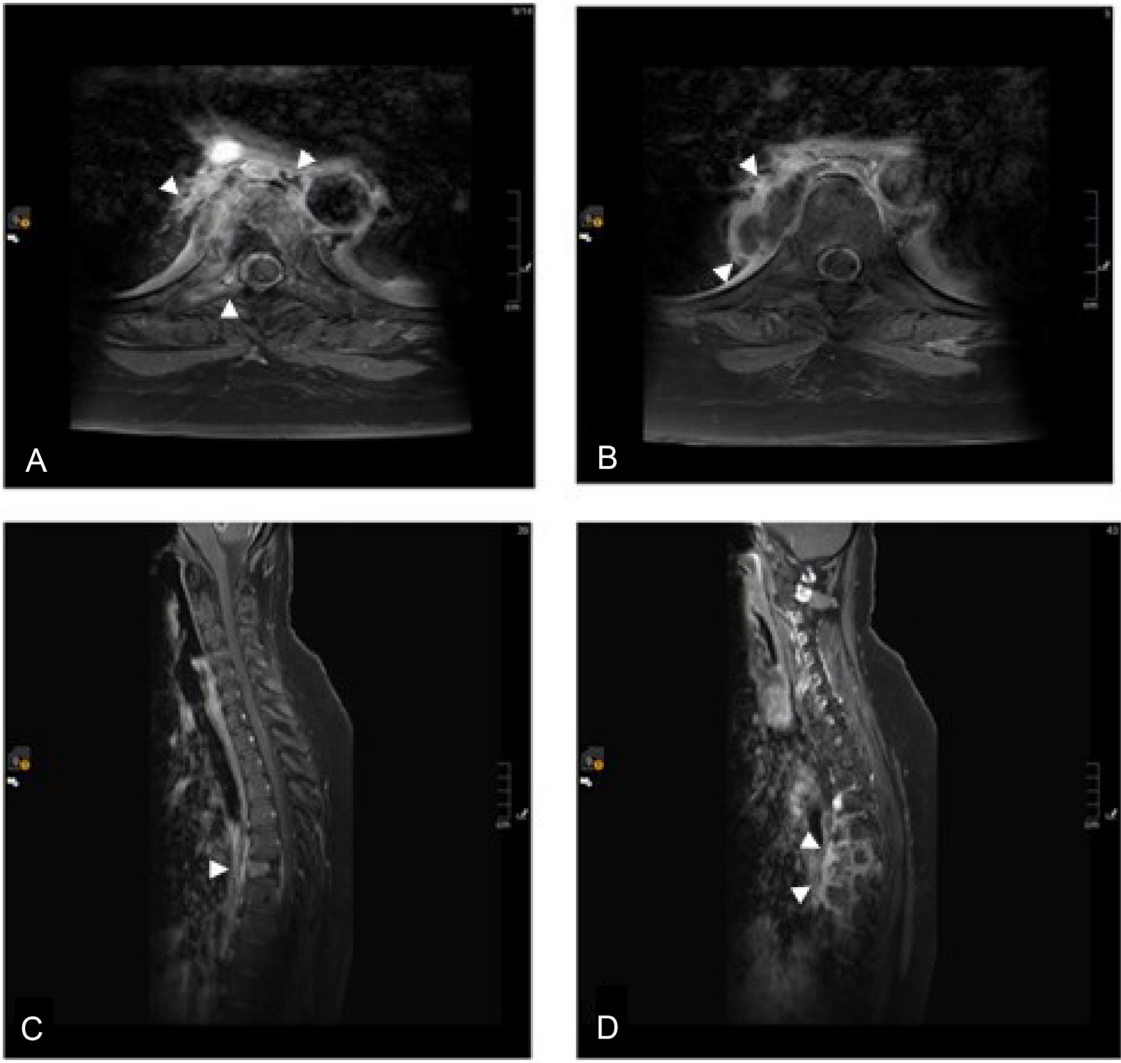
Axial (A and B) and sagittal (C and D) post gadolinium MRI of the thoracic spine showing infiltrative osseous lesion involving T6 vertebral body, extending to the right pedicle and transverse process sparing the adjacent disks, with right paravertebral peripherally enhancing collection extending from T5-T7 levels (arrow heads).

A contrasted Computed Tomography (CT) chest was performed as part of the initial diagnostic work-up. The scan revealed new intraosseous air locules within the sixth thoracic vertebral body, accompanied by a right paravertebral collection measuring 3.7×1.5 cm. The air locules were observed to extend into the right paravertebral region and right pleura (Figs. 1 and 2). Subsequently, a whole spine Magnetic Resonance Imaging (MRI) with contrast was conducted, which revealed an enhancing infiltrative osseous lesion involving the T6 vertebral body. The lesion extended to the right pedicle and right transverse process, causing compression of the right exiting nerve root and a low-grade epidural spinal cord compression (grade 1c). Additionally, a septated paravertebral lesion on the right side was observed, indicating the presence of an infected collection measuring 4×2 cm in axial dimensions and 5.7 cm in craniocaudal dimensions. Signal intensity alterations and enhancement were also detected in the posterior aspect of the right sixth rib, suggesting the presence of osteomyelitis. These findings collectively suggested T6 osseous metastasis associated with underlying emphysematous osteomyelitis and a paravertebral abscess (Fig. 3).

CT-guided aspiration of the paravertebral collection was performed, and the aspirated material was sent for culture. The results revealed the growth of Salmonella enterica subspecies, which corresponded with the blood culture results obtained at the time of admission, confirming the presence of the same microorganism. Based on the sensitivity results, the patient was maintained on ceftriaxone at a recommended dose of 2 mg administered twice daily, as advised by the infectious diseases team.

After 11 days of antibiotic treatment, a follow-up spine CT scan was conducted, which showed mild interval sclerosis and improvement in the intraosseous gas locules at the T6 level. However, a larger, more well-defined right paravertebral fluid collection was observed. To address this collection, a CT-guided drain insertion procedure was performed in the right paravertebral region. Eight days after drain insertion (19 days after antibiotic treatment initiation), the CRP level decreased to 5 mg/L (normal range: less than 8-10 mg/L), indicating a positive response to treatment, and the drain was removed.

However, the treatment was not yet complete. The patient later developed upper thoracic back pain again, and MRI revealed enlargement of a metastatic lesion in the T6 vertebral body, resulting in grade 2 epidural spinal cord compression. Considering the patient's overall poor medical condition, a recommendation was made for palliative external beam radiotherapy (EBRT) targeting the T6 region. The patient subsequently underwent palliative radiotherapy over T6, receiving a total dose of 20 Gray administered in 5 fractions.

Unfortunately, the patient's disease progression was uncontrollable, and a diagnosis of lymphangitis carcinomatosis was made. Additionally, the patient's brain metastasis worsened, resulting in vasogenic edema, leading to a decline in consciousness and subsequent aspiration pneumonia. These factors further deteriorated the patient's condition, ultimately leading to her death 70 days after her initial presentation. No autopsy was performed.

### Case 2

**Fig. 4:**
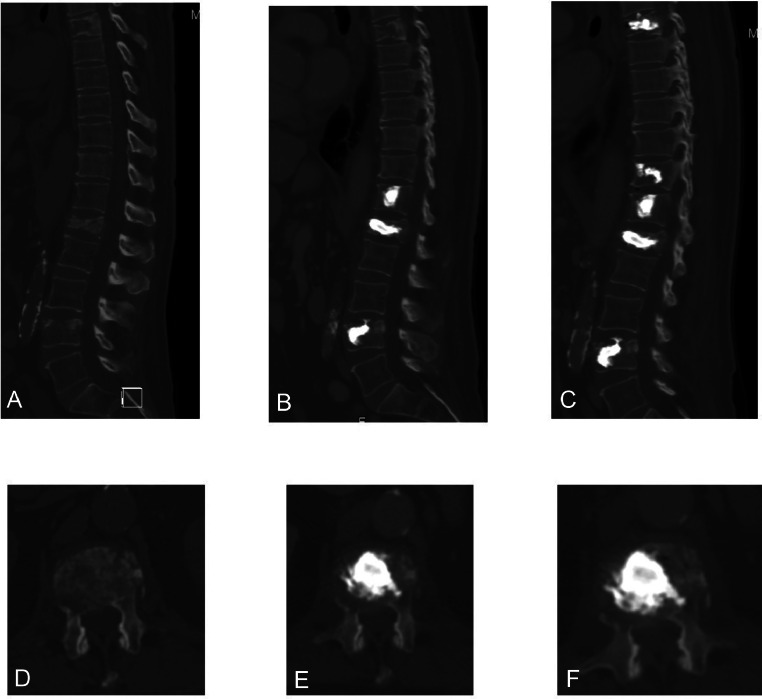
Sagittal (A, B, and C) and axial (D, E, F) showing CT scan findings at presentation (A and D), 2 weeks after surgery (B and E) and 6 weeks after surgery (C and F).

The second case involves a 66-year-old male patient who was diagnosed with de novo metastatic lung adenocarcinoma to the bone and bone marrow. The patient had no known chronic medical conditions. He initially presented to the spine clinic 1 month after the diagnosis, complaining of mechanical back pain concentrated in the upper thoracic and thoracolumbar areas. MRI and CT scans were performed, which showed multiple metastatic lesions involving the T6, T11, T12, L1, L4, and S1 vertebrae. The patient also had a pathologic compression fracture at L1. The case was discussed in the spine multidisciplinary tumor board, and a decision was made to perform extra-pedicular kyphoplasty for the L1 fracture and cement augmentation for T6, T11, T12, and L4, which were done in 2 stages (Fig. 4).

**Fig. 5:**
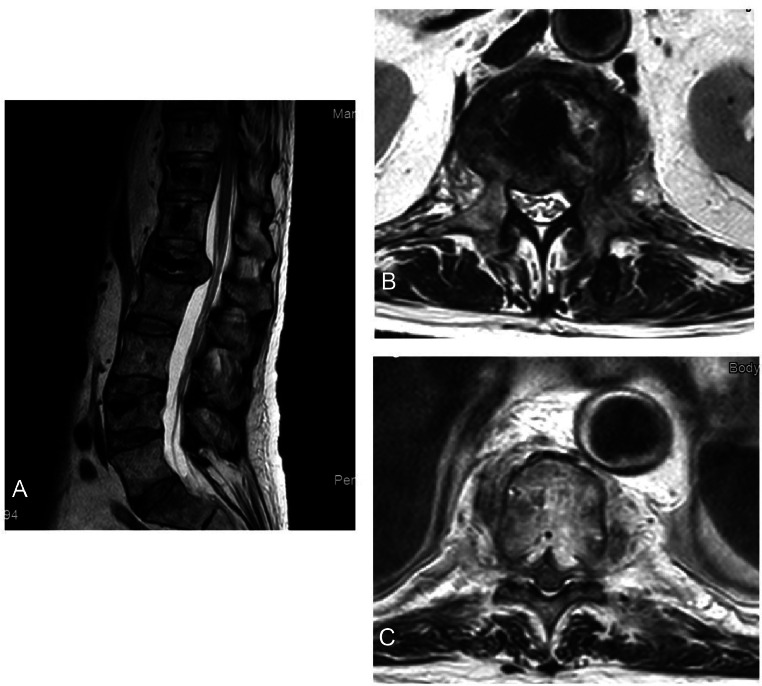
Sagittal (A) and axial (B and C) contrast enhanced MRI of the thoracolumbar spine 6 weeks after surgeries showing infiltrative osseous lesions involving L1 and T10 vertebral body with soft tissue component compression the cord at L1 (B) and at T10 (C).

Following the procedures, the patient initially did well, but later started complaining of severe mechanical back pain and pain at rest. A follow-up MRI and CT scan were conducted 6 weeks after surgery, revealing mild progression in the L1 fracture over the cement, with a soft tissue component compressing the spinal cord at L1 and the T10 vertebrae. Palliative cord decompression and posterior fixation surgery were recommended; however, the patient refused the procedure due to the high associated mortality and morbidity (Fig. 5).

Consequently, the patient underwent palliative radiotherapy targeting the T10-L1 area, receiving a total dose of 20 Gray administered in 5 fractions. Over 6 weeks after radiotherapy, the patient's condition progressively worsened, leading to admission to the hospital for pain control. Notably, the patient's basic metabolic panel (BMP) and white blood cell (WBC) count were within normal limits.

Upon admission, the patient was started on intravenous (IV) morphine for pain management. He also received dexamethasone to reduce inflammation and edema around the tumor. A consultation with the palliative care team was made to optimize pain control and provide supportive care**.**

**Fig. 6:**
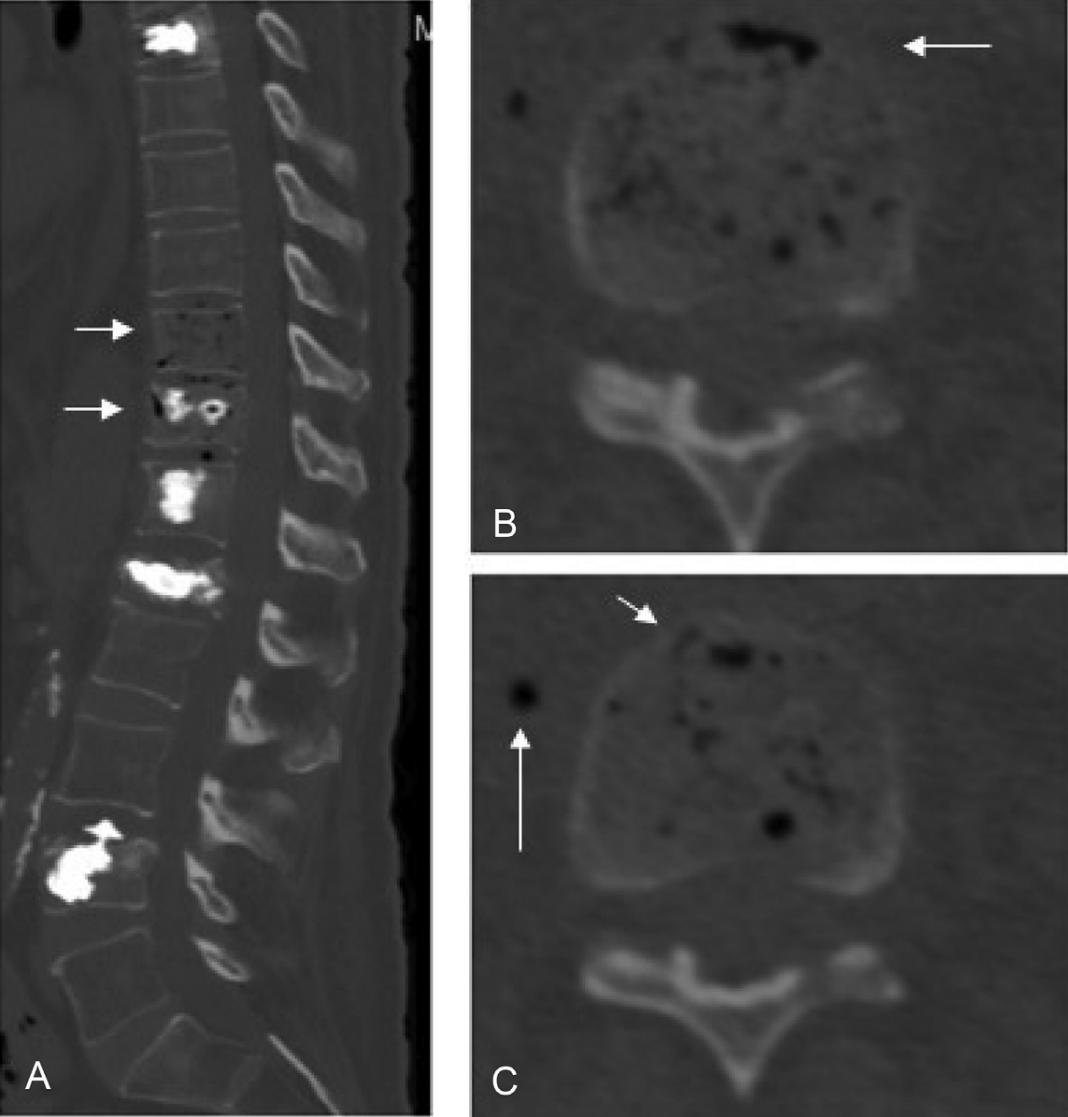
(A) sagittal view of the thoracic spine showing air locules inside the T9 and T10 vertebral bodies (white arrows). (B) and (C) axial view of the thoracic spine showing intraosseous air locules inside the T9 vertebral body (short arrow) associated with extension of the air locules into the right paravertebral region (long arrow).

Because the patient experienced intense and incapacitating pain that required a significant amount of narcotics to manage, surgical intervention was once again presented as an option. In addition, follow-up MRI and CT scans were conducted to assist in preoperative preparation. The CT scan revealed characteristic indications of EO, including the presence of gas bubbles within the T10 and T11 bodies (Fig. 6).

The CT scan findings raised suspicion of EO, prompting further investigation. Blood cultures, urine cultures, ESR, and CRP tests were conducted, and a CT-guided biopsy was performed. The CRP level was notably elevated at 287, and the cultures revealed the presence of Staphylococcus saprophyticus. The patient was initially placed on broad-spectrum antibiotics but later switched to vancomycin alone for 6 weeks, based on the culture results. Subsequently, the patient experienced significant improvement in symptoms, the pain was effectively managed, and they were able to resume walking with support.

## Discussion

The first identification of intraosseous gas as a symptom of osteomyelitis dates back to 1981 [3]. When intraosseous gas is observed in the axial skeleton, it is primarily associated with noninfectious causes. However, the presence of substantial intravertebral gas, bone edema, or related collections suggests a higher probability of osteomyelitis, which was evident in our patients [4].

Only a limited number of documented cases (25 in total) have been reported in the existing literature regarding emphysematous osteomyelitis (EO) affecting both the axial and appendicular skeleton. Our literature strategy was to search for case reports, case series, or systematic reviews containing emphysematous osteomyelitis regardless of specific sites. Among these cases, only one instance involved Salmonella enterica subspecies causing EO in both femur heads through hematogenous spread. Additionally, no cases have been reported thus far where Staphylococcus saprophyticus was the causative agent. Therefore, we didn't find a previously reported case represents the initial report of EO affecting the vertebrae caused by Salmonella enterica subspecies, while the second case represents the first reported case of EO involving the vertebrae caused by Staphylococcus saprophyticus.

The primary mode of transmission for monomicrobial infections is hematogenous spread, while continuous spread is typically observed in polymicrobial infections. As in the first case, diabetes mellitus was frequently observed as a comorbidity, consistent with the majority of reported cases [4]. Both patients were on a high-dose corticosteroid treatment, which could potentially have a significant impact on the infection, particularly in cancer patients who may be frequently exposed to such medications.

Prompt evaluation, diagnosis, and early treatment could prevent dismal outcomes if delay occurs [5]. The most effective test for diagnosing vertebral infections is MRI, which has recorded sensitivity and specificity of 96% and 93%, respectively [6]. In both cases, the diagnosis was made through CT scans rather than MRI.

**Fig. 7:**
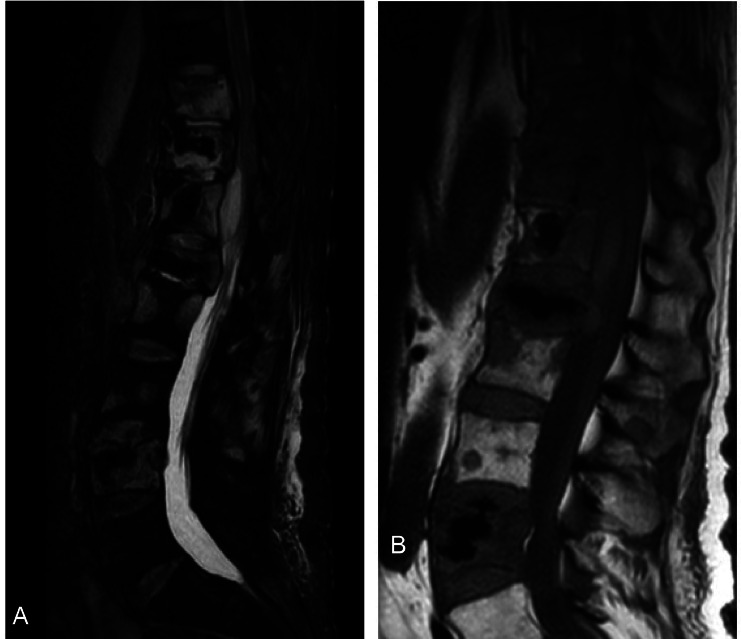
Sagittal T2 (A) and T1 (B) contrast enhanced MRI of the thoracolumbar spine showing similar enhancement between the infected vertebrae (T9 and T10) and the metastatic lesions distal to that.

The MRI scans displayed almost typical findings in both the infected vertebrae and metastatic lesions (Fig. 7). This highlights the importance of utilizing both imaging modalities, CT and MRI, in cases of suspected osteomyelitis associated with metastatic tumors in the spine. Ideally, it is recommended to perform aspiration and cultures at the site of infection if a collection is identified. Blood cultures should also be obtained. Furthermore, it is mandatory to initiate broad-spectrum antibiotics that provide coverage against aerobic and anaerobic bacteria, with subsequent adjustments based on the results of the culture. In cases where fluid collection is detected at the site of infection, surgical intervention or image-guided drainage may be considered.

## Conclusions

Emphysematous osteomyelitis (EO) is a rare and potentially life-threatening condition that necessitates prompt diagnosis. The preferred imaging modalities for diagnosing EO are CT and MRI. Therefore, a comprehensive radiological assessment is crucial in suspected cases. Based on our specific experience, we have observed that recognizing and identifying the typical CT findings is essential, as it enables timely initiation of antibiotics and appropriate drainage procedures, either through surgical intervention or image-guided techniques. Early identification and treatment can significantly reduce morbidity in this vulnerable patient population and may improve prognosis.

## Authors' contributions

The authors confirm their contribution to the paper as follows: data collection: A.Alelaumi and I.Althunibat; analysis and; draft manuscript preparation: All authors. All authors reviewed the results and approved the final version of the manuscript.

## Statements and declarations

Ethical approval was waived by the ethics committee in King Hussein Cancer Center as the research is a retrospective study involving less than 4 patients.

## Ethical approval

Ethical approval was waived by the ethics committee in King Hussein Cancer Center, Amman, Jordan.

## Data availability

The data that supports the findings of this study are available in the supporting information of this article.

## Patient consent

Written informed consent was obtained from the patients for publication and any accompanying images.
